# High adenoviral vector concentration can cause irreversible aggregation during CsCl gradient ultracentrifugation

**DOI:** 10.3389/fmicb.2026.1845210

**Published:** 2026-06-04

**Authors:** Haidong Wu, Ya-Fang Mei

**Affiliations:** Department of Clinical Microbiology, Virology, Umeå University, Umeå, Sweden

**Keywords:** adenovirus vectors, chemical restoration, EM morphology, high concentration, irreversible aggregation, OD calculation, ultracentrifugation, virus loading

## Abstract

Highly purified adenovirus preparations are essential for fundamental research and therapeutic use. However, the impact of viral concentration during CsCl gradient ultracentrifugation on virion integrity is poorly understood, and this incomplete mechanistic understanding may adversely affect product quality and constrain further advances in research. In this study, we examined how viral input affects adenovirus infectivity, cytotoxicity, yield, and structural integrity using transmission electron microscopy and analysis tools. High-concentration preparations (AdV-HC) showed significant aggregation, capsid damage, and considerably lower TCID₅₀ values, while optimally concentrated preparations (AdV-OC) preserved intact icosahedral structures and high infectivity. Efforts to reverse aggregation failed, showing that excessive viral concentration during purification causes irreversible structural harm. These findings indicate that overloading during CsCl ultracentrifugation promotes vector aggregation, thereby compromising virion stability and biological activity. To preserve structural integrity and ensure consistent performance, we recommend maintaining the final purified vector concentration at or below 2 mg/mL. Based on this threshold, we defined theoretical optimal optical density (OD) ranges for adenovirus concentration in cell lysates prior to ultracentrifugation to minimize vector aggregation during purification. Therefore, this study provides a simple and effective strategy for improving the consistency, purity, and infectivity of adenovirus preparations for both research and clinical applications.

## 1 Introduction

Adenoviruses are widely used as application platforms for gene delivery, vaccine development, and oncolytic virotherapy because of their high transduction efficiency, broad tropism, and capacity for genetic modification (Wong et al., 2012; Zhang et al., 2018). Species B adenoviruses, including type 11p, have become promising alternatives to the commonly used Ad5 vectors due to their improved ability to transduce hematopoietic stem cells and other clinically relevant cell types, as well as their reduced susceptibility to pre-existing immunity (Shayakhmetov et al., 2000; Mei et al., 2004). These qualities have made adenovirus a therapeutic application (Dyer et al., 2017; Wu and Mei, 2019).

Despite these advantages, producing high-quality adenoviral preparations remains a significant technical challenge. Many downstream applications demand viral stocks with high purity, structural integrity, and infectivity. CsCl gradient ultracentrifugation is a common method for adenovirus purification because it efficiently separates intact virions from empty capsids and cellular contaminants by buoyant density (Wilcox and Ginsberg, 1963). However, the effects of upstream parameters, especially viral load, on particle integrity and functional quality during ultracentrifugation remain poorly understood.

Viral aggregation is a well-known phenomenon that can diminish infectivity and stability in many viruses (Kahler et al., 2016; Pradhan et al., 2022). Both reversible and irreversible aggregation processes are influenced by physicochemical conditions, including ionic strength, buffer composition, and macromolecular interactions (Haag et al., 2002; Rexroad et al., 2006; Stepanenko et al., 2021). With adenoviruses, aggregation can occur during production, purification, or storage, but the mechanisms and functional impacts are poorly understood (Galdiero, 1979). It is unclear whether chemical treatments can reduce aggregation-induced defects or if they cause irreversible structural damage to the viral capsid (Jackson and Mantsch, 1991; Arakawa et al., 2007).

During the investigation of replication-competent adenovirus 11p vectors, we occasionally observed a loss of infectivity following ultracentrifugation. This effect was restricted to genetically modified constructs, including vectors with insertions of the adenovirus death protein (ADP) or cytokine genes into either the E1 or E3 region (Mei et al., 2016; Wu and Mei, 2019). This intriguing phenomenon has drawn considerable attention, as an incomplete understanding of its mechanisms may compromise product quality and limit further advances in research. To elucidate this virus inactivation problem, we systematically examined here how viral load influences adenovirus aggregation during CsCl gradient ultracentrifugation and its effects on viral infectivity and structural integrity. We show that high viral concentrations lead to aggregation that cannot be reversed by common chemical agents, resulting in a significant loss of biological activity despite intact viral DNA. Additionally, we identify vector-specific factors, such as genome insertion size, that affect susceptibility to aggregation.

## 2 Materials and methods

### 2.1 Adenovirus vector construction

The Ad11p vector used in this study is protected by intellectual property rights (Patent No. EP 2 486 137 B1). In this study, the replication-competent Ad11p vector was modified to include an inserted DNA segment (about 1,450 base pairs) placed upstream of the E3 region. Even though the cassette contains the CMV promoter, a cytokine gene, and SV40 poly A, its biological activity is not relevant here.

### 2.2 Cell lines and culture conditions

A549 cells (a human lung carcinoma cell line) were used to propagate the Ad11p vector. Cells were maintained in Dulbecco’s Modified Eagle Medium (DMEM) supplemented with 5% fetal bovine serum, 20 mM HEPES, and 1% penicillin–streptomycin at 37 °C.

### 2.3 Virus preparation and vector quantification

The viruses were propagated in A549 cells, which were harvested 3.5–4 days post-infection (Gokumakulapalle and Mei, 2016). Cells were pelleted in 50 mL Falcon tubes by centrifugation at 600 RPM, approximately 18 x g for 5 min at 4 °C using an Eppendorf centrifuge equipped with an A-4-62 rotor and washed once with 12 mL of 20 mM Tris–HCl (pH 7.5). The cell pellet was resuspended in PBS and lysed by sonication (two 10 s pulses at 30% power on ice). Virions were extracted by adding an equal volume of chloroform, then manually shaking for 2–3 min. The extract was then centrifuged at 3000 rpm for 2 min, and the upper phase was collected. A total of 6 mL/each of clarified extract was layered onto a discontinuous CsCl density gradient consisting of 2.5 mL of 1.27 g/mL CsCl, 2 mL of 1.32 g/mL CsCl, and 1.5 mL of 1.37 g/mL CsCl, arranged from top to bottom. The gradient was centrifuged at 25,000 rpm (approximately 107,000 × g) for 2h at 4 °C using a Beckman SW41 rotor. The resulting virion band was collected, and its buoyant density was determined using a refractometer. Virus concentration was measured spectrophotometrically by calculating the absorbance at 260 nm minus that at 330 nm, where one optical density (OD) unit corresponds to 280 μg/mL or 1 × 10^12^ virus particles.

### 2.4 Virus titration and cytopathic effect assay

Virus infectivity was assessed by determining the tissue culture infectious dose (TCID₅₀) in A549 cells. A total of 1.5 × 10^4^ cells were seeded per well in 96-well plates and incubated overnight. Serial tenfold dilutions of virus (ranging from 3.6 × 10^4^ to 3.6 × 10^−2^ VPs/cell) were applied. CPE was observed daily, and titers were calculated. TCID₅₀ values were determined using the Reed–Muench method, a standard approach for estimating infectious viral titers based on endpoint dilution assays (Reed and Muench, 1938).

### 2.5 Toxicity assay

To evaluate oncolytic effects, A549 cells were seeded in 24-well plates and infected with serial dilutions of adenovirus vector in optimal concentration.

(AdV-OC) and adenovirus vector in high concentration (AdV-HC; 3.6 × 10^4^ to 3.6 × 10^−2^ VPs/cell). After 8 days post-infection (p.i.), cytotoxicity was assessed using crystal violet staining. Cells were fixed with 4% paraformaldehyde (PFA) for 3 min, washed with phosphate-buffered saline (PBS), stained with 1% crystal violet in 70% ethanol for 3 min, and rinsed thoroughly before air-drying and imaging (Sandberg et al., 2009).

### 2.6 Restoring the AdV-HC function viruses with NaCl and/or DMSO in PBS

AdV-HC viruses were treated with single chemicals or a combination of two chemicals to restore their function (Arakawa et al., 2007). 2-fold dilution of NaCl, 0.125–1 M, or DMSO, 1.56–12.5% in PBS buffer. The mixture contained NaCl and DMSO, each at half their respective concentrations, with the actual concentration ranges as follows: NaCl, from 0.0625 M to 0.5 M; DMSO, from 0.78 to 6.25%. AdV-HC was incubated in the solution for 2 h at RT, then diluted in PBS, and its infectivity was measured in a 96-well plate. Serial dilutions of the virus were prepared from 10^−1^ to 10^−9^ VPs/cell.

### 2.7 Negative staining and transmission electron microscopy

Highly concentrated viral stock (10,229 μg/mL; Table 1) was evaluated after proper dilution in ultrapure water. 3 μL of AdV-OC or AdV-HC at the same concentration was applied to glow-discharged, carbon-coated, Formvar-supported 400-mesh copper grids (Ted Pella) and incubated for ~30 s to allow particle adsorption. Excess liquid was carefully blotted using filter paper. The grids were subsequently washed briefly with ultrapure water to remove residual buffer components. Negative staining was performed by applying 2% (w/v) uranyl acetate for contrast enhancement, followed by blotting and air-drying. Imaging was carried out using a Hitachi HT7700 transmission electron microscope (Hitachi High-Technologies) operated at an accelerating voltage of 80 kV. Micrographs were recorded with a 2k × 2k Veleta CCD camera (EMSIS GmbH).

**Table 1:** Comparison of the OD, yield of two viral samples after CsCl gradient ultracentrifugation.

Sample	Volume (mL)	O. D	Dilution (fold)	Total (OD/mL)	Conc. (mg/mL)	Conc. (VPs/mL)	Yield (mg/1.5mL)	Yield (VPs/1.5mL)
AdV-HC	1.5	0.365	100	36.5	10.220	3.65 × 10^13^	15.330	5.475 × 10^13^
AdV-OC	1.5	0.332	20	6.64	1.859	8.32 × 10^12^	2.789	1.248 × 10^13^

The table presents the A260 OD values corrected for background absorbance at A330. Virus concentration was measured spectrophotometrically by calculating the absorbance at 260 nm minus that at 330 nm, where one optical density (OD) unit corresponds to 280 μg/mL or 1 × 10^12^ adenovirus particles.

## 3 Results

### 3.1 Clear CsCl gradient bands and distinct yields of AdV-OC and AdV-HC preparations

In this study, a replication-competent adenovirus serotype 11p (AdV-11p) vector was generated by insertion of an expression cassette encoding a 1,450 bp therapeutic gene. High viral yields were obtained following propagation in A549 cells. The complete cell lysate was subsequently divided into two fractions prior to ultracentrifugation: a 20% fraction representing four T175 cm^2^ flasks (AdV-OC) and an 80% fraction representing sixteen T175 cm^2^ flasks (AdV-HC).

**Figure 1:**
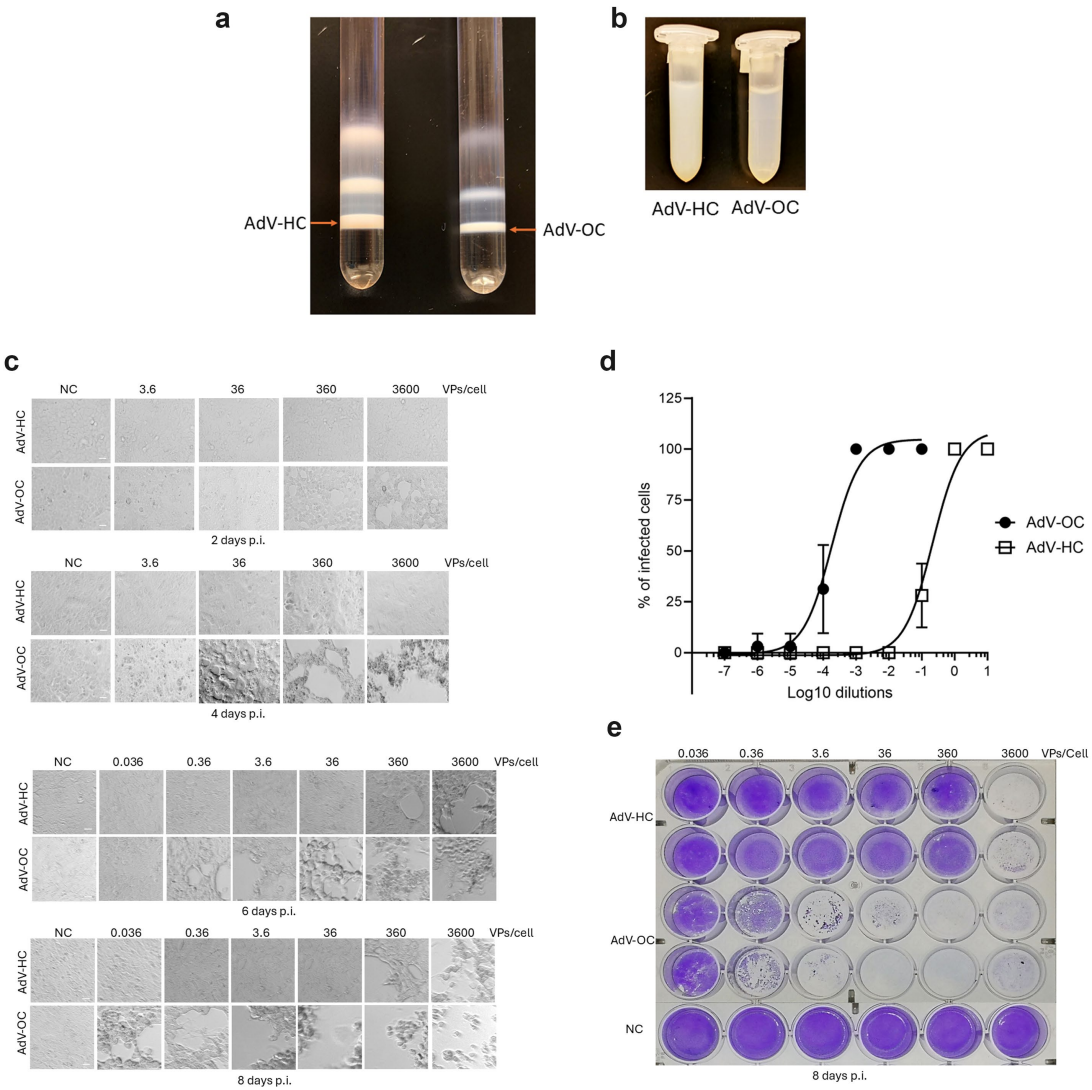
Comparison of adenovirus banding, infectivity, and cytopathic effect of AdV-HC and AdV-OC. **(a, b)** Viral bands following CsCl gradient ultracentrifugation of high-concentration (AdV-HC) and optimally concentrated (AdV-OC) adenovirus preparations. Distinct, well-defined viral bands are indicated. **(c)** Cytopathic effect (CPE) of AdV-HC and AdV-OC in A549 cells. Cells were infected with varying viral particle numbers (0.0036–3600 VPs/cell, corresponding to 0.00001–1 pg. virus/cell) and incubated for 2, 4, 6, and 8 days post-infection (p.i.). CPE was monitored daily using light microscopy at 200 × magnification. Representative images are shown. The white scale bar represents 15 μm and was generated in Fiji. **(d)** Quantification of the percentage of infected cells across the range of viral doses. Data represent the mean ± SD from at least three independent experiments. **(e)** Histogram comparing infection efficiency and cytotoxicity mediated by AdV-HC and AdV-OC in A549 cells.

Following ultracentrifugation, both fractions exhibited distinct and well-defined viral bands without visible smearing or detectable cellular debris, indicating efficient purification. The primary observable difference between the two preparations was band intensity, consistent with differences in viral density. No visible precipitation was observed in either preparation, suggesting comparable macroscopic purity. Quantitative analysis revealed that both optical density and total viral yield of AdV-HC were approximately 5.5-fold higher than those of AdV-OC (Table 1; Figures 1a,b), regardless of whether expressed as mg/mL or viral particles per millilitre (VPs/mL). Importantly, this distinction between intact infectious virions and nonfunctional or aggregated particles is addressed by electron microscopy in the following section.

### 3.2 Viral concentration critically influences the biological properties of adenoviruses following ultracentrifugation

Functional characterization revealed striking differences in biological activity between the two preparations. AdV-OC induced a pronounced cytopathic effect (CPE) in A549 cells as early as 24 h post-infection (p.i.), whereas AdV-HC produced minimal infection, with delayed evidence of CPE. CPE progression was monitored every second day up to 8 days p.i. At this time point, AdV-OC was CPE-positive at a 10^−5^ dilution (0.036 VPs/cell), whereas AdV-HC showed reduced CPE at a 10^−1^ dilution (360 VPs/cell; Figures 1c–e). Infectivity was further quantified by determining the 50% tissue culture infectious dose (TCID₅₀) in A549 cells. The ratio of infectious particles to total viral particles (IP: VP) for AdV-OC fell within the expected range (corresponding to ≥1:72), whereas AdV-HC failed to meet the acceptable threshold (<1:72). Based on these criteria, only AdV-OC preparations were considered suitable for downstream validation experiments.

### 3.3 Electron microscopy reveals aggregation-associated capsid disruption

**Figure 2:**
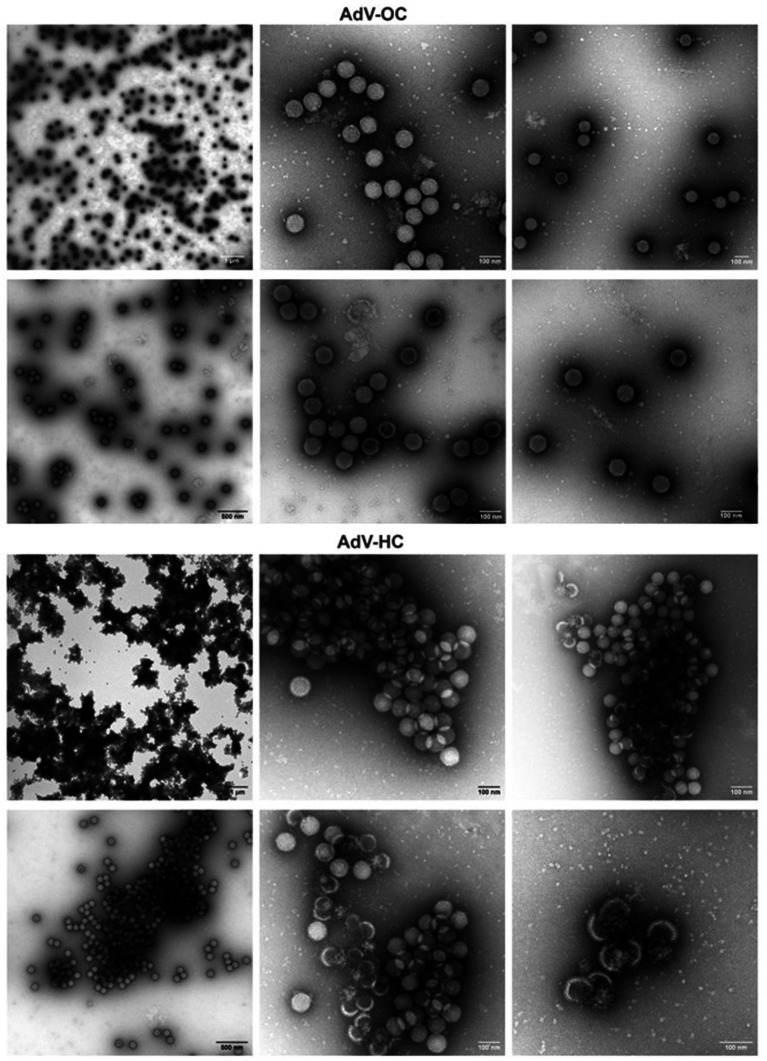
Transmission electron microscopy reveals ultrastructural features of AdV-OC and AdV-HC. Morphology of adenovirus preparations was assessed by negative staining and transmission electron microscopy. AdV-HC was analyzed either as a concentrated preparation or after dilution with ultrapure water, and AdV-OC was analyzed under identical conditions. Samples were prepared on grids and stained with 2% uranyl acetate following routine procedures. Micrographs reveal the structural features of the virions, with all particles appearing morphologically intact and free from detectable cellular debris, protein aggregates, or extraneous DNA. Representative images are shown. White or black scale bars indicate the corresponding distances and were generated using Fiji software.

The structural basis underlying the functional differences between AdV-HC and AdV-OC was investigated by electron microscopy. AdV-HC was diluted to 1 μg/μL as AdV-OC’s concentration, after negative staining with 2% (w/v) uranyl acetate. AdV-HC exhibited extensive particle aggregation, capsid interpenetration, and large super-aggregates formed by tightly compressed virions (Figure 2). These observations indicate that high-concentration preparations undergo irreversible structural failure, characterized by capsid collapse and inter-virion fusion incompatible with productive infection. In contrast, TEM revealed that AdV-OC particles were uniformly sized, retained their intact icosahedral morphology, and displayed well-defined capsid edges, with no aggregation observed across the fields examined (Figure 2). These observations indicate that the high-density preparation is associated with more pronounced capsid structural disruption than the optimally concentrated virus preparation.

### 3.4 Aggregation-induced capsid damage is chemically irreversible

Given the extensive aggregation observed by electron microscopy, we next examined whether chemical treatment could restore the functionality of aggregated AdV-HC particles. Attempts to recover AdV-HC using sodium chloride (NaCl, 0.125–1 M) or dimethyl sulfoxide (DMSO, 1.56–12.5%), applied individually or in combination, to restore viral function. Following these measures, the infectivity of the restored viruses and of untreated AdV-OC and AdV-HC was used as high- and low-infectivity viral control, respectively.

**Figure 3:**
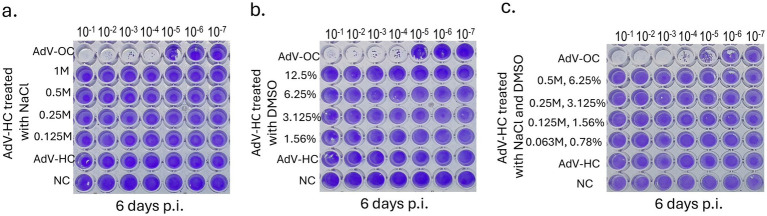
Chemical strategies for restoring aggregated AdV-HC using NaCl, DMSO, or combined treatment. AdV-HC virus samples were treated under varying concentrations of NaCl (0.125–1M), dimethyl sulfoxide (DMSO, 1.56–12.5% v/v), or a combination of both (NaCl, 0.0625–0.5M; DMSO, 0.78–6.25% v/v) to evaluate potential recovery of viral function. **(a)** Salt-only treatments were used to assess the effects of ionic strength on capsid stabilization; **(b)** DMSO-only treatments were applied to probe the influence of protein conformation; **(c)** Combination treatments tested matched concentrations of salt and DMSO to identify potential synergistic or antagonistic effects. Restoration efficiency was quantified by measuring infectious titers (TCID_50_/mL) and normalized to untreated AdV-HC and AdV-OC controls. Results demonstrate that chemical treatment failed to restore infectivity, indicating that the aggregation-induced structural damage is irreversible.

Consistent with these findings, highly aggregated AdV-HC exhibited markedly reduced infectivity, with infectious titers approximately four orders of magnitude lower than those of AdV-OC (Figure 3). Collectively, results from failed-to-disaggregate experiments demonstrate that excessive viral concentration during purification promotes adenovirus aggregation, thereby indicating irreversible capsid damage and a profound loss of infectivity.

### 3.5 Theoretical calculation of vector concentrations in cell lysates from vector yields

The true initial OD is calculated from the total recovered OD (Table 1), corrected for recovery efficiency:


$$\text{True initial}\;\mathrm{OD}=\frac{\text{Total recovered}\;\mathrm{OD}}{\text{Recovery efficiency}}$$

*AdV-OC*: Total recovered OD = 6.64 × 1.5 = 9.96 OD. A recovery efficiency of 70% is assumed.


$$\text{True total}\;\mathrm{OD}=\frac{9.96}{0.7}=14.23$$

Normalizing to the original lysate volume (6 mL):


$$\text{True initial}\;\mathrm{OD}=\frac{14.23}{6}=2.37\;(\mathrm{OD}/\mathrm{mL})$$

*AdV-HC*: Total recovered OD = 36.5 × 1.5 = 54.75 OD. A recovery efficiency of 70% is assumed.


$$\text{True total}\;\mathrm{OD}=\frac{54.75}{0.7}=78.21$$

Normalizing to the original lysate volume (6 mL):


$$\text{True initial}\;\mathrm{OD}=\frac{78.21}{6}=13.04\;\;(\mathrm{OD}/\mathrm{mL})$$

Therefore, based on the true total yield of harvested viral vectors and assuming a 70% recovery rate, we calculated both the true initial total viral OD and the OD per unit volume for AdV-HC and AdV-OC preparations prior to ultracentrifugation, yielding estimated values ranging from 2.37 to 13.04 OD/mL. Under an alternative assumption of 50% recovery, the estimated true initial total viral OD values for AdV-OC and AdV-HC increased to 3.32 and 18.25 OD/mL, respectively. These estimates indicate substantial differences in viral concentrations within the cell lysates, with markedly higher viral loads in AdV-HC than in AdV-OC preparations. Notably, a certain number of cellular proteins, DNA, and RNA must be included in the true initial virus OD. Usually, with proper sample preparation, the amount of cellular material present in the cell lysate is much lower than that of the virus. These findings further suggest that quantification of cell lysates prior to ultracentrifugation may be valuable under certain conditions, as it can help assess sample loading and reduce the risk of vector aggregation or overloading during downstream purification.

## 4 Discussion

This study presents an improved method for producing adenovirus preparations that are pure, stable, and highly infectious, and identifies viral load as a critical determinant of preparation quality. After cells are infected, fully lysed, and debris is removed, the viral concentration must be carefully controlled before purification by CsCl density gradient ultracentrifugation. Too high a concentration causes irreversible aggregation, while optimized concentrations consistently produced intact, infectious virus particles. In addition, based on the yield of the purified vector, we derived a theoretical optimal OD value for the vector in the cell lysate preparations. This reference value may help control viral loading and minimize vector aggregation and inactivation during ultracentrifugation in future preparations. Notably, in addition to reflecting high viral vector content, cell lysates must exhibit several characteristic features for OD measurements to be considered meaningful and reliable. These include typical cytopathic effects (CPE) associated with viral infection, formation of a well-defined cell pellet, and a uniform light milky-white appearance of the lysate. In contrast, lysates that appear viscous, excessively transparent, or flocculent generally indicate poor sample quality, making OD measurements unreliable or uninterpretable.

Viral concentration is the primary driver of aggregation, but other physicochemical factors can also play a role (Rexroad et al., 2006). Aggregation has been observed across a wide range of viruses, both RNA and DNA, enveloped and non-enveloped, and can be triggered by variations in buffer composition, pH, salt concentration, peptide sequence, and chemical treatments (Galdiero, 1979; Kahler et al., 2016; Pradhan et al., 2022). These factors can significantly affect how infectious and biologically active a virus is. In cell culture, adenoviral particles can stick to host DNA. This can sometimes be reversed by increasing the sodium chloride concentration, which helps separate the particles and restore infectivity.

A different type of aggregation was reported in a study of the Ad5RGD4C vector, which carries a modified fiber knob HI domain (Stepanenko et al., 2021). During the second CsCl gradient centrifugation step, visible aggregations formed within the gradient, and the preparation yielded less virus with a narrower viral band than control samples. In this case, a peptide (4C) inserted into the HI domain of the fiber knob likely caused the particles to cross-link, possibly through covalent bonds formed under the stress of ultracentrifugation, resulting in irreversible aggregation driven by the high particle density. This type of structurally driven aggregation is fundamentally different from what we observed in our study.

Highly aggregated AdV-HC preparations exhibited infectious titers approximately three orders of magnitude lower than those of non-aggregated AdV-OC, indicating a profound loss of biological activity. Importantly, these findings demonstrate that optical absorbance-based quantification of viral DNA is insufficient to assess functional integrity in aggregated samples. Direct infectivity assays are therefore essential for accurate evaluation of viral potency.

Taken together, our data indicate that aggregation induces irreversible structural damage to the viral capsid that is refractory to chemical restoration. These findings underscore the importance of preventing aggregation during virus purification to maintain viral integrity. We therefore recommend implementing preventive measures after cell lysis to ensure optimal gradient-loading conditions.

Vector-specific properties further influence aggregation propensity. Notably, a replication-competent adenovirus 11p vector carrying a 1,450 bp expression cassette exhibited a pronounced tendency to aggregate during CsCl ultracentrifugation, whereas vectors containing larger inserts (>1,729 bp; e.g., GFP or RFP) remained stable under identical conditions (Mei et al., 2016). This observation suggests that genome size and insertion length may affect capsid stability and inter-particle interactions, potentially enhancing resistance to aggregation at high particle densities.

Species B adenovirus type 11p vectors offer distinct advantages over other adenoviral platforms, including high productivity and favorable structural stability. The strategy described here enables the reliable production of high-quality adenovirus preparations suitable for both experimental and potential therapeutic applications. While the present study is limited to a subset of vectors and insert sizes, future work will extend these findings to a broader range of constructs and further elucidate the mechanistic basis of adenoviral aggregation.

## Data Availability

The raw data supporting the conclusions of this article will be made available by the authors, without undue reservation.

## References

[ref1] Arakawa T. Kita Y. Timasheff S. N. (2007). Protein precipitation and denaturation by dimethyl sulfoxide. Biophys. Chem. 131, 62–70. doi: 10.1016/j.bpc.2007.09.004, 17904724

[ref2] Dyer A. Di Y. Calderon H. Illingworth S. Kueberuwa G. Tedcastle A. . (2017). Oncolytic group B adenovirus enadenotucirev mediates non-apoptotic cell death with membrane disruption and release of inflammatory mediators. Mol Ther Oncolytics 4, 18–30. doi: 10.1016/j.omto.2016.11.003, 28345021 PMC5363721

[ref3] Galdiero F. (1979). Adenovirus aggregation and preservation in extracellular environment. Arch. Virol. 59, 99–105. doi: 10.1007/BF01317899, 34378

[ref4] Gokumakulapalle M. Mei Y. F. (2016). Replication-competent human adenovirus 11p vectors can propagate in Vero cells. Virology 495, 42–51. doi: 10.1016/j.virol.2016.04.029, 27176913

[ref5] Haag L. Garoff H. Xing L. Hammar L. Kan S. T. Cheng R. H. (2002). Acid-induced movements in the glycoprotein shell of an alphavirus turn the spikes into membrane fusion mode. EMBO J. 21, 4402–4410. doi: 10.1093/emboj/cdf442, 12198142 PMC126182

[ref6] Jackson M. Mantsch H. H. (1991). Beware of proteins in Dmso. Biochim. Biophys. Acta 1078, 231–235. doi: 10.1016/0167-4838(91)90563-F, 2065090

[ref7] Kahler A. M. Cromeans T. L. Metcalfe M. G. Humphrey C. D. Hill V. R. (2016). Aggregation of adenovirus 2 in source water and impacts on disinfection by chlorine. Food Environ Virol 8, 148–155. doi: 10.1007/s12560-016-9232-x, 26910058 PMC4864101

[ref8] Mei Y. F. Segerman A. Lindman K. Hornsten P. Wahlin A. Wadell G. (2004). Human hematopoietic (CD34+) stem cells possess high-affinity receptors for adenovirus type 11p. Virology 328, 198–207. doi: 10.1016/j.virol.2004.07.018, 15464840

[ref9] Mei Y. F. Wu H. Hultenby K. Silver J. (2016). Complete replication-competent adenovirus 11p vectors with E1 or E3 insertions show improved heat stability. Virology 497, 198–210. doi: 10.1016/j.virol.2016.07.026, 27494367

[ref10] Pradhan S. Varsani A. Leff C. Swanson C. J. Hariadi R. F. (2022). Viral aggregation: the knowns and unknowns. Viruses 14, 438–461. doi: 10.3390/v14020438, 35216031 PMC8879382

[ref11] Reed L. J. M. Muench H. (1938). A simple method of estimating fifty percent endpoints. Am. J. Hyg. 27:5.

[ref12] Rexroad J. Evans R. K. Middaugh C. R. (2006). Effect of pH and ionic strength on the physical stability of adenovirus type 5. J. Pharm. Sci. 95, 237–247. doi: 10.1002/jps.20496, 16372304

[ref13] Sandberg L. Papareddy P. Silver J. Bergh A. Mei Y. F. (2009). Replication-competent Ad11p vector (RCAd11p) efficiently transduces and replicates in hormone-refractory metastatic prostate cancer cells. Hum. Gene Ther. 20, 361–373. doi: 10.1089/hum.2007.124, 19199789

[ref14] Shayakhmetov D. M. Papayannopoulou T. Stamatoyannopoulos G. Lieber A. (2000). Efficient gene transfer into human CD34(+) cells by a retargeted adenovirus vector. J. Virol. 74, 2567–2583. doi: 10.1128/JVI.74.6.2567-2583.2000, 10684271 PMC111745

[ref15] Stepanenko A. A. Sosnovtseva A. O. Valikhov M. P. Chekhonin V. P. (2021). A new insight into aggregation of oncolytic adenovirus Ad5-delta-24-RGD during CsCl gradient ultracentrifugation. Sci. Rep. 11:16088. doi: 10.1038/s41598-021-94573-y, 34373477 PMC8352973

[ref16] Wilcox W. C. Ginsberg H. S. (1963). Structure of type 5 adenovirus. I. Antigenic relationship of virus-structural proteins to virus-specific soluble antigens from infected cells. J. Exp. Med. 118, 295–306. doi: 10.1084/jem.118.2.295, 14074393 PMC2137710

[ref17] Wong H. H. Jiang G. Gangeswaran R. Wang P. Wang J. Yuan M. . (2012). Modification of the early gene enhancer-promoter improves the oncolytic potency of adenovirus 11. Mol. Ther. 20, 306–316. doi: 10.1038/mt.2011.242, 22086234 PMC3272483

[ref18] Wu H. Mei Y. F. (2019). An oncolytic adenovirus 11p vector expressing adenovirus death protein in the E1 region showed significant apoptosis and tumour-killing ability in metastatic prostate cells. Oncotarget 10, 1957–1974. doi: 10.18632/oncotarget.26754, 30956777 PMC6443017

[ref19] Zhang W. W. Li L. Li D. Liu J. Li X. Li W. . (2018). The first approved gene therapy product for Cancer ad-p53 (Gendicine): 12 years in the clinic. Hum. Gene Ther. 29, 160–179. doi: 10.1089/hum.2017.218, 29338444

